# R-Spondin 2 and WNT/CTNNB1 Signaling Pathways Are Required for Porcine Follicle Development and In Vitro Maturation

**DOI:** 10.3390/ani11030709

**Published:** 2021-03-05

**Authors:** Seon-Ung Hwang, Junchul David Yoon, Mirae Kim, Lian Cai, Hyerin Choi, Dongjin Oh, Eunhye Kim, Sang-Hwan Hyun

**Affiliations:** 1 Laboratory of Veterinary Embryology and Biotechnology (VETEMBIO), College of Veterinary Medicine, Chungbuk National University, Cheongju 28644, Korea; ghkdsun@hanmail.net (S.-U.H.); jdyoon86@gmail.com (J.D.Y.); kmr9309@naver.com (M.K.); cailian002@daum.net (L.C.); hyrin3642@naver.com (H.C.); rosecafes123@naver.com (D.O.); 2 Institute of Stem Cell & Regenerative Medicine (ISCRM), Chungbuk National University, Cheongju 28644, Korea

**Keywords:** porcine, in vitro maturation, cumulus cell, WNT/CTNNB1 pathway, RSPO2

## Abstract

**Simple Summary:**

A recent mouse study reported that R-spondin2 promotes follicular development by activating WNT/Catenin beta-1 (CTNNB1) signaling in granulated cells. Our results demonstrate that RSPO2, CTNNB1, G-protein coupled receptor 4 (LGR4), and G-protein coupled receptor 5 (LGR5) factors play a major role in porcine follicular development, and epidermal growth factor receptor (EGFR) signaling is essential for in vitro maturation. The R-spondin2 and WNT/CTNNB1 signaling pathways are involved in porcine follicle development, and it is expected that the EGFR-ERK signaling pathway is also involved.

**Abstract:**

The secretion of oocyte-derived paracrine factors, such as R-spondin2, is an essential mechanism for follicle growth by promoting the proliferation and differentiation of cumulus cells around oocytes. In the present study, we aimed to identify the effect of R-spondin2 during follicular development. First, R-spondin2-related factors (R-spondin2, CTNNB1, LGR4, and LGR5) were identified through immunofluorescence in porcine ovarian tissue. CTNNB1 was expressed in ooplasm, and CTNNB1 and LGR4 were expressed in granulosa cells. In addition, R-spondin2, LGR4, and LGR5 were expressed in the theca interna. These results imply that these proteins play a major role in porcine follicular development. In addition, the effects of R-spondin2 on the in vitro maturation process of porcine cumulus oocyte complexes and subsequent embryonic development were confirmed. A treatment of 100 ng/mL R-spondin2 in the in vitro maturation (IVM) process increased nuclear maturation and increased the expression of *EGFR* mRNA in cumulus cells. The EGFR-ERK signal is essential for oocyte maturation, ovulation, and luteinization. R-spondin2 treatment also increased the expression of CTNNB1 and EGFR in primary cultured cumulus cells. In conclusion, RSPO2 and WNT/CTNNB1 signaling pathways are required for porcine follicle development and are predicted to be involved in the EGFR-ERK signaling pathway.

## 1. Introduction

In vitro maturation (IVM) is a widely used model that effectively mimics the growth of follicles in vivo. The secretion of oocyte-derived paracrine factors is essential for follicle growth by promoting the proliferation and differentiation of the cumulus cells around oocytes. At least three oocyte-derived factors have been identified to date to promote the growth of granulosa cells: roof plate-specific spondin2 (RSPO2), growth differentiation factor-9, and bone morphogenic protein-15 [1]. Recently, both Growth differentiation factor-9 (GDF9) and RSPO2 have been found to be essential for granulosa cell proliferation: RSPO2 activates WNT/Catenin Beta 1 (CTNNB1) signaling, and GDF9 signaling has been reported to act through the Bone morphogenetic protein receptor type II (BMPR2)/Activin receptor type-1B (ALK4) heterodimer receptor and the Mothers against decapentaplegic homolog 2/3 (SMAD2/3) pathway in granulosa cells [2,3].

All RSPO protein family members require the WNT ligand and low-density lipoprotein receptor-related protein 6 (LRP6) for their activity and amplify the signaling of WNT3A, WNT1, and WNT7A [4]. RSPO2 is one of the four distinct members of the RSPO protein family (RSPO1–4), which show amino acid identities greater than 40% to 60% and are expected to exhibit structural homology [5].

RSPO2 promotes the development of primary and secondary follicles and drives progression to the maturation stage in mice in vivo. The effect of RSPO2 on follicles mimics the effect of follicle-stimulating hormone (FSH), but with a distinct intracellular mechanism of signal transduction [6]. In rodents, the FSH and WNT signaling pathways were shown to activate the WNT signaling-related molecule CTNNB1 (also known as β-catenin) in granulosa cells to enhance FSH action and promote pre-ovarian follicular growth and survival. However, excessive expression of CTNNB1 inhibits the downstream response to the luteinizing hormone (LH)-induced terminal differentiation of granulosa cells [7,8]. In addition, the concentration of RSPO2 in human follicular fluid correlates with the number of retrieved and mature metaphase II (MII) stage oocytes. Thus, RSPO2 may provide new therapeutic options for infertile women with a poor response to traditional gonadotropin therapy [9].

The canonical WNT/CTNNB1 signaling pathway transmits signals through CTNNB1 in the cytoplasm and regulates target gene expression [10]. It is the main pathway for communication between cells (paracrine) or within cells (self-secreting). Without WNT, CTNNB1 does not accumulate in the cytoplasm because it is broken down by disruptive complexes such as axin, adenomatosis polyposis coli (APC), protein phosphatase 2A (PP2A), glycogen synthase kinase 3 (GSK3), and casein kinase 1 α (CK1α) [11,12]. The canonical WNT/CTNNB1 signal is activated when a WNT ligand in the extracellular space associated with the cell membrane binds to the transmembrane FZD receptor that cooperates with LRP5/6 [13,14]. Subsequently, the protein complex of APC, GSK3B, and Axin is destroyed, and CTNNB1 accumulates in the cytoplasm [15]. The accumulated CTNNB1 is translocated to the nucleus, which controls transcriptional regulation through binding to the T-cell factor/lymphoid enhancer binding protein (TCF/LEF) [16,17]. Several factors involved in the WNT signaling pathway have been reported to directly, or indirectly, modulate the development of follicles [18,19,20,21]. Studies with mouse follicles showed that treatment with the WNT signal activators LiCl and WNT3a increased the proportion of abnormal follicles, whereas treatment with the WNT inhibitor IWR-1 significantly increased the ratio of normal follicles [22]. In contrast, in mouse granulosa cells, WNT2 regulates DNA synthesis by acting through β-catenin downstream of FZD9 [23], which is important for follicle growth as it plays a role in maintaining gap–junction communication within the follicle [24]. In addition, WNT2 signals through Frizzled Class Receptor 9 (FZD9) to regulate the β-catenin pathway in human cumulus cells; thus, the WNT2/CTNNB1 signal plays an important role in human folliculogenesis [25]. In pigs, when Dickkopf-related protein 1 (DKK1) was administered at the in vitro culture stage, the hatchability of the blastocysts (BLs) and the number of cells per BL increased, whereas LiCl treatment decreased both the number of cells and the hatchability of BLs. Thus, WNT/CTNNB1 signaling is likely to be involved in the hatching and trophectoderm differentiation of porcine blastocysts [26]. In addition, another study showed that the addition of DKK1 increased the proportion of mature oocytes to MII and consequently increased the number of nuclei in the BL stage [27].

Epidermal growth factor receptor (EGFR) has been reported to play an important physiological role in cell survival, proliferation, adhesion, invasion, motility, and angiogenesis, and is a member of the ErbB family of transmembrane receptor tyrosine kinases [28,29,30]. EGFR can be activated by EGF-like peptides (e.g., HB-EGF, TGF-α, AREG, and EREG) to promote several signaling pathways, including JAK/STAT, PI3K, and MAPK pathways [31,32,33]. Recent studies have reported that EGFR induces the expansion of granulosa and cumulus cells in follicles and promotes cytoplasmic maturation, meiosis resumption, and ovulation, by transmitting LH signals to oocytes [34,35]. RSPO2 binds to leucine-rich repeat-containing G-protein coupled receptor 4 or 5 (LGR4 or 5), which induces the transcription of matrix metalloproteinase 9 (*MMP9*) by increasing the activity of WNT/CTNNB1. MMP9 secretes an activated form of heparin-binding epidermal growth factor (HB-EGF), that further activates EGFR-ERK signaling to induce progesterone secretion and corpus luteum maturation in granulosa-lutein cells [36].

According to this background, the RSPO2 protein is a common regulator of canonical WNT signaling through the activation of β-catenin. However, studies on the relationship between RSPO2 and the WNT signaling pathway during follicular development have thus far been limited to rodents. Therefore, there is a need to understand the functional role of RSPO2 in the formation of follicles in other mammals, and to determine the molecular mechanisms that regulate the WNT signaling pathway. Because porcine models are optimized for IVM, it is easy to study the related signaling pathways during oocyte maturation. Therefore, the aim of the present study was to evaluate the relationship between RSPO2 and the WNT pathway during porcine follicular development. To this end, oocytes and COCs were treated with human recombinant RSPO2, LiCl, or DKK1, during IVM, and evaluated for their effects on WNT signaling, apoptosis, proliferation, and expression of genes associated with RSPO2. In addition, in vitro fertilization (IVF) experiments were performed to evaluate the effect of RSPO2-WNT signaling on embryo development.

## 2. Materials and Methods

### 2.1. Chemicals

Recombinant Human R-Spondin 2 (3266-RS-025; R&D System, Minneapolis, MN, USA) was dissolved in 0.1% (wt/vol) Dulbecco’s phosphate-buffered saline (DPBS)-bovine serum albumin (BSA). The solution was stored at −70 °C until it was used in the experiment. All of the other chemicals were purchased from Sigma-Aldrich Chemical Company (St. Louis, MO, USA).

### 2.2. Immunofluorescence (IF) for Paraffin-Embedded Sections

The ovaries with small (1 to 2 mm), medium (3 to 6 mm), and large (7 to 9 mm) follicles were treated in 10% formalin for 24–48 h at room temperature (RT). The dissected tissue (less than 3 mm thick) was placed in a tissue cassette, and then rinsed with running tap water for 10 min. After that, the manufacture of paraffin blocks and the paraffin section slides were commissioned by Chungbuk National University Laboratory Animal Center (Chungju, Korea). Prepared tissue slides were incubated for 30 min in a 60 °C drying oven. The slides were deparaffinized with 2 changes of xylene, for 5 min each, and then transferred to 100%, 90%, 80%, and 70% EtOH, for 5 min each. The tissue was then rinsed with running tap water for 10 min. For antigen retrieval, 10 mM sodium citrate buffer (pH 6.0) was preheated using a microwave, placed into a 100 °C water bath, and then boiled. The slides were placed in a preheated citrate buffer for 20 min, before being removed and allowed to cool at RT for 20 min. The slides were then rinsed 2 times in Tris-buffered saline (TBS-T, pH 7.4) for 5 min each.

Moisture was then removed from the slides, and a border was drawn around the tissue with a PAP Pen as a hydrophobic barrier (ImmEdge™; Vector Laboratories, Burlingame, CA, USA). The slides were then placed in a humid chamber before being washed twice for 5 min with TBS-T, and treated in blocking solution (10% goat serum in PBS) for 2 h at RT. The slides were then rinsed 3 times in TBS-T for 5 min each. The primary antibody and blocking solution were then co-incubated overnight at 4 °C. Antibodies used in this study are listed in Table 1. On the following day, the slides were washed 3 times with TBS-T for 5 min each and incubated with the appropriate secondary antibodies at RT for 1 h. The slides were then rinsed 3 times in TBS-T for 5 min each, and nuclei were stained with DAPI, before being washed three times with TBS-T for 5 min each and mounted with an anti-fade mounting medium (Molecular Probes, Inc., Eugene, OR, USA). The stained organoid sections were then examined by confocal laser microscopy (Carl Zeiss, Thornwood, NY, USA). All of the images were analyzed with the ZEN blue edition software.

### 2.3. Oocytes Collection and IVM

Porcine ovaries were collected from the slaughterhouse, placed in a thermos containing 37 °C, 0.9% (wt % vol) NaCl solution, and transferred to the laboratory. Using an 18G needle and syringe, porcine follicular fluid (pFF) and cumulus oocyte complexes (COCs) were aspirated in medium 3–6 mm follicles. After COCs were selected under a stereoscopic microscope, approximately 50–60 COCs per well were transferred to a Nunc 4-well dish (Nunc, Roskilde, Denmark) with 500 μL of maturation medium. The composition of the maturation medium was 0.6 mM cysteine, 0.91 mM sodium pyruvate, 10 ng/mL EGF, 75 μg/mL kanamycin, 1 μg/mL insulin, and 0.1% (wt/vol) polyvinyl alcohol (PVA) in TCM 199 (Gibco, Grand Island, New York, USA). RSPO2 was used to treat the COCs during IVM at concentrations of 0, 0.5, 5, 10, and 100 ng/mL. The WNT inhibitor (recombinant human DKK-1, 120-30; Peprotech, NJ, USA) was treated at a concentration of 200 ng/mL, and the WNT activator (LiCl) was treated at a concentration of 15 mM. The solvent used in the stock preparation was 0.1% BSA + dPBS. For the first 22 h after IVM, 10 IU/mL equine chronic gonadotropin (eCG) and 10 IU/mL human chorionic gonadotropin (hCG) (Intervet, Boxmeer, The Netherlands) were treated in a maturation medium. Thereafter, eCG was not treated for 20 h. IVM was performed at 39 °C in 5% CO_2_ using a humid incubator (Astec, Fukuoka, Japan). After 42 h of the start of IVM, mature COCs were exposed to gentle pipetting with 0.1% hyaluronidase and TLH-PVS medium. Matured cumulus cells and oocytes were used for subsequent experiments.

### 2.4. Fluorescence Staining for Assessment of Nuclear Maturation

Matured oocytes were stained with 10 µg/mL Hoechst 33342 dye, and nuclear maturation was assessed using a fluorescence microscope (TE300, Nikon, Tokyo, Japan) and a micromanipulator (NT-88-V3, Narishige, Japan). Nuclear maturation was evaluated by classifying cells into four stages (germinal vesicle, metaphase I, anaphase and telophase I, and metaphase II).

### 2.5. Gene Expression Analysis Quantitative Polymerase Chain Reaction (qPCR)

Matured cumulus cells and oocytes were sampled individually and stored at −80 °C until the experiment. RNA extraction and cDNA synthesis were performed by using a SuperPrep™ Cell Lysis & RT Kit for qPCR (Toyobo Co., Osaka, Japan) according to the manufacturer’s protocol. qPCR mixtures were prepared using synthesized cDNA and 2× SYBR Premix Ex Taq (Takara Bio, Inc., Otsu, Shiga, Japan) with primers synthesized by Macrogen (Seoul, Korea). All of the primer sequences are presented in Table 2. qPCR was performed on an Mx3000P qPCR instrument (Agilent Technologies, Santa Clara, CA, USA). The reactions were performed for 40 cycles, and the cycling parameters were as follows: denaturation at 95 °C for 30 s, annealing at 57 °C for 30 s, and extension at 72 °C for 30 s. Relative quantification was based on a comparison of the threshold cycle (Ct) at constant fluorescence intensity. The relative mRNA expression (R) was calculated by using the equation R = 2^−[ΔCt sample−ΔCt control]^. To determine the normalized arbitrary value for each gene, every value was normalized to that of *RN18S* [37].

### 2.6. Cumulus Cell Culture

The cumulus cell culture medium was composed of Dulbecco’s modified eagle medium (DMEM) containing 10% FBS, 1% non-essential amino acids, 1% glutamine, 0.1 mM β-mercaptoethanol, and 1% antibiotics-antimycotics (all from Gibco). Cumulus cells were cultured at 37 °C in a 5% CO_2_ humid incubator (Astec).

### 2.7. In Vitro Fertilization (IVF) and In Vitro Culture (IVC)

IVF was performed as previously described [38]. We purchased and used fresh liquid semen (Xperm-V, Darby Genetics Inc., Anseong, Korea) within 3 days of collection. Briefly, IVF was performed by selecting mature oocytes in the MII stage. MII oocytes were washed thrice by using TLH-PVA, and 15 oocytes were transferred to modified Tris-buffered medium (mTBM) droplets. The final concentration of sperm was diluted to 5 × 10^5^/mL and co-cultured with the oocytes at 39 °C in a 5% CO_2_ humid incubator for 20 min. The sperm on the surface of embryos were detached by gentle pipetting and moved to an mTBM drop. Embryos were cultured for 5 h and then incubated at 39 °C in a 5% CO_2_/O_2_ humid incubator with 10 embryos per droplet in 25 μL of porcine zygote medium 3 (PZM3) droplets for IVC [39]. On the second day after fertilization, embryo cleavage (CL) was evaluated (1-cell, 2 to 3-cell, 4 to 5-cell, and 6 to 8-cell stages, and fragmented embryos) and transferred to new PZM3 droplets. On the fourth day, the embryos were transferred to PZM3 droplets containing 10% FBS. On the seventh day after fertilization, embryo developmental performance was quantitatively assessed by the CL rate and BL formation rate (early, expanded, and hatched blastocysts). All droplets were covered with mineral oil.

### 2.8. Statistical Analysis

Statistical analyses were carried out using SPSS 21.0 (Statistical Package for the Social Sciences, Inc., Chicago, IL, USA). Rates of nuclear maturation, embryo development data (e.g., CL, BL formation, and total cell number of BL), and relative mRNA expression levels were compared by one-way ANOVA (Analysis of variance), followed by Duncan’s multiple range test. Data are reported as mean ± SEM. Differences were considered significant if the *p*-value was less than 0.05.

### 2.9. Experimental Design

#### 2.9.1. Experiment I. Localization and Expression of RSPO2-Related Genes under Normal Conditions

IF staining was first performed on the expression site of RSPO2-related factors according to the estrous cycle of the porcine ovaries. Thereafter, the maturation time (0, 22, and 42 h) was sampled to determine the gene expression pattern in oocytes and cumulus cells. After extracting the total RNA from each sample, cDNA was synthesized by reverse transcription. The relative mRNA expressions were confirmed by qPCR. The genes used in this experiment were WNT (*WNT3a*, *CTNNB1*, *EGFR*, *Follistatin*) and RSPO2 (*RSPO2*, *LGR4*, *LGR5*) signaling related genes.

#### 2.9.2. Experiment II. The Effect of R-Spondin 2 in IVM with Porcine Oocytes According to Different Concentrations

This experiment was performed to determine the optimal concentration of RSPO2 in the IVM process. RSPO2 was treated in COCs for IVM at concentrations of 0, 0.5, 5, 10, and 100 ng/mL. After 42 h IVM, nuclear maturation was evaluated.

#### 2.9.3. Experiment III. Confirmation of the Mechanism of R-Spondin 2 IVM of Porcine Cumulus-Oocyte Complexes

To confirm the mechanism by which RSPO2 promotes the IVM process, COCs were treated with RSPO2 (100 ng/mL), the WNT inhibitor (DKK1; 200 ng/mL), or the WNT activator (LiCl; 15 mM) during IVM. The Dkk1 concentration was the same as in the paper reported in 2014 by Spate et al. [27]. LiCl has been reported to be effective above 5 mM in pigs IVM [40] and there has been no toxic effect up to 20 mM of LiCl [41]. Nuclear maturation was evaluated 42 h after the start of IVM, and the expression levels of WNT signaling (*CTNNB1*, *EGFR*), RSPO2 (*RSPO2*, *LGR5*), cell proliferation (*PCNA*), cumulus cell expansion (*PTX3*) [42], pluripotency (*POU5F1*), and apoptosis (*BAX*, *BCL2*) related genes were determined by qPCR in mature oocytes and cumulus cells as described above.

Next, IVF was performed using matured oocytes to confirm embryo developmental performance before implantation. Embryo developmental performance was assessed by CL rate and BL formation rate. Matured oocytes used for IVF were treated with RSPO2 (100 ng/mL), WNT inhibitor (DKK1; 200 ng/mL) or WNT activator (LiCl; 15 mM) during IVM, and not treated at the IVC stage.

#### 2.9.4. Experiment IV. Confirmation of the Mechanism of R-Spondin 2 in Porcine Cumulus Cells

Under general IVM conditions, only mature cumulus cells were isolated and cultured in the cell culture medium. After sufficient proliferation, the cumulus cells were treated with the same concentration of RSPO2, WNT inhibitor Dkk1, and WNT activator LiCl. The gene expression levels of the primary cultured cumulus cells were determined by qPCR for WNT signaling (*CTNNB1*, *EGFR*), *RSPO2*, cell proliferation (*PCNA*), and cumulus cell expansion (*HAS2*, *PTX3).*

## 3. Results

### 3.1. Identification and Localization of RSPO2 Related Factors in Ovarian Tissues According to Follicle Size

**Figure 1:**
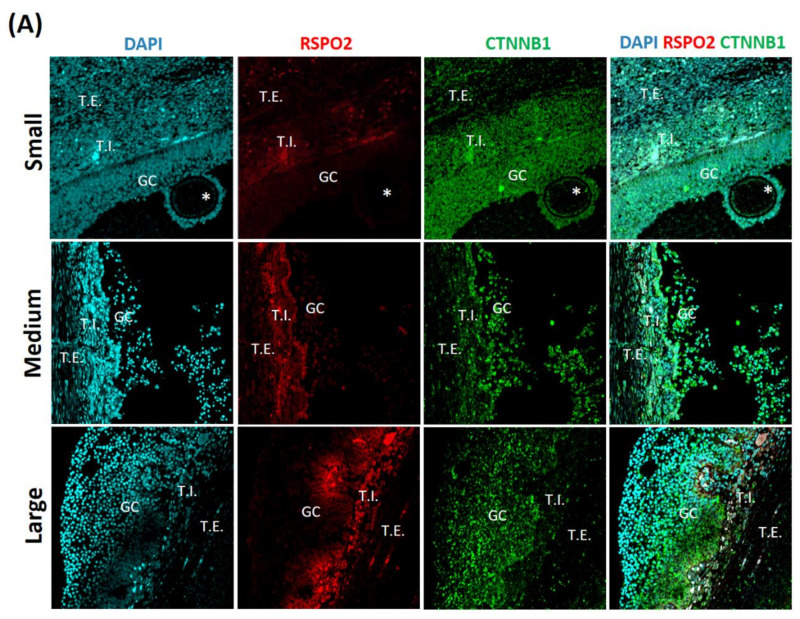

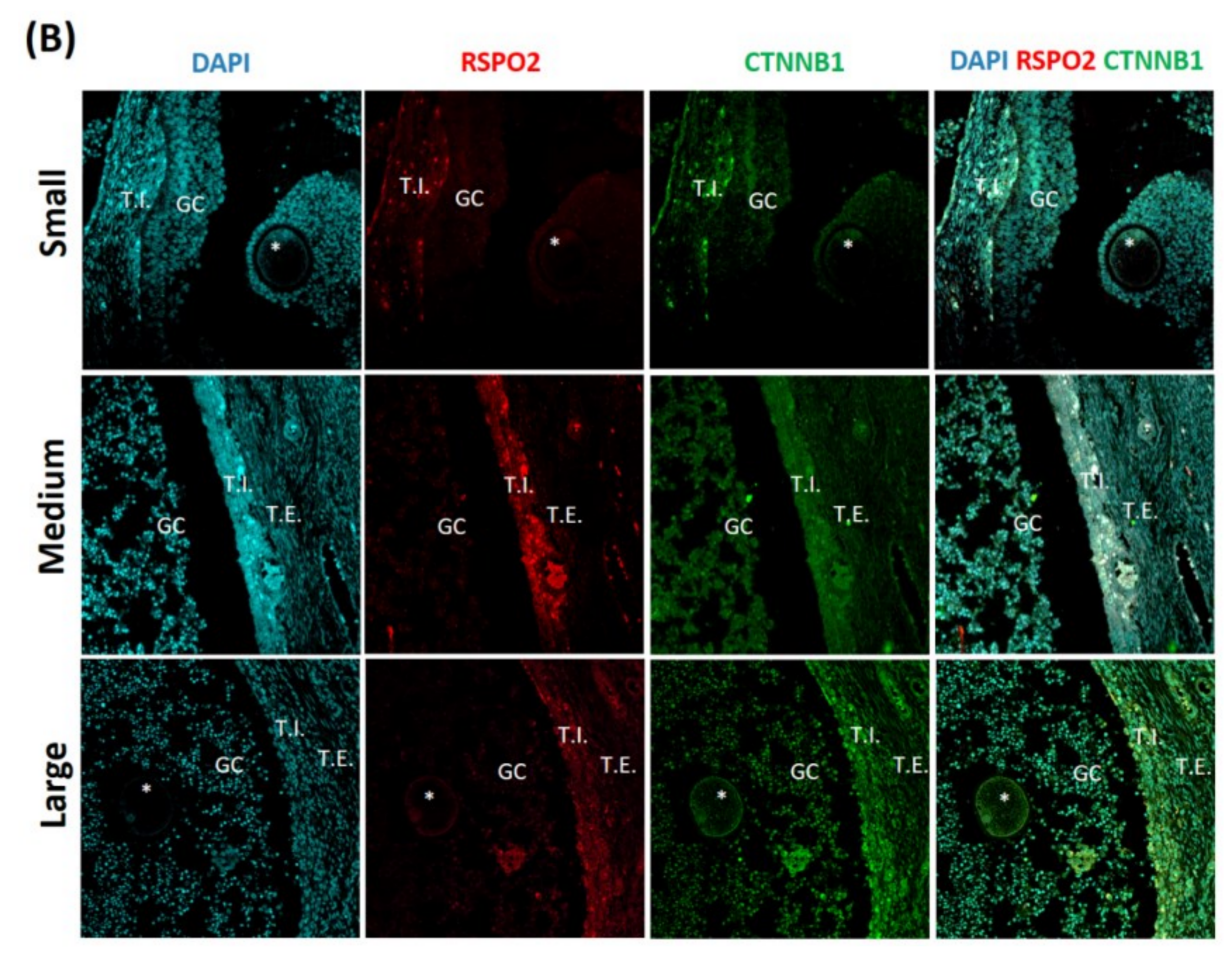
Identification and localization of roof plate-specific spondin2 (RSPO2) related factors in porcine ovarian tissues according to follicle size by immunofluorescence. Each ovary with small (1–2 mm), medium (3–6 mm), and large (7–9 mm) follicles. (**A**) RSPO2 and Catenin beta-1 (CTNNB1), (**B**) G-protein coupled receptor 4 (LGR4) and G-protein coupled receptor 5 (LGR5). theca interna (T.I.), theca externa (T.E.), granulosa cells (GC), and ooplasm (*).

To gain an insight into the role of RSPO2 in the estrus cycle of porcine ovaries, we first identified the expression position of the RSPO2 related factors (RSPO2, CTNNB1, LGR4, and LGR5) by the size of the porcine ovarian follicles (small, medium, and large). RSPO2 was mainly identified in theca interna (T.I.), and CTNNB1 was expressed in Granulosa cells and ooplasm. LGR4 was identified in T.I. and Granulosa cells, and LGR5 was expressed in T.I. There was no difference according to follicle size (Figure 1).

### 3.2. Expression of mRNA in Cumulus Cells and Oocytes According to Maturation Time

**Figure 2:**
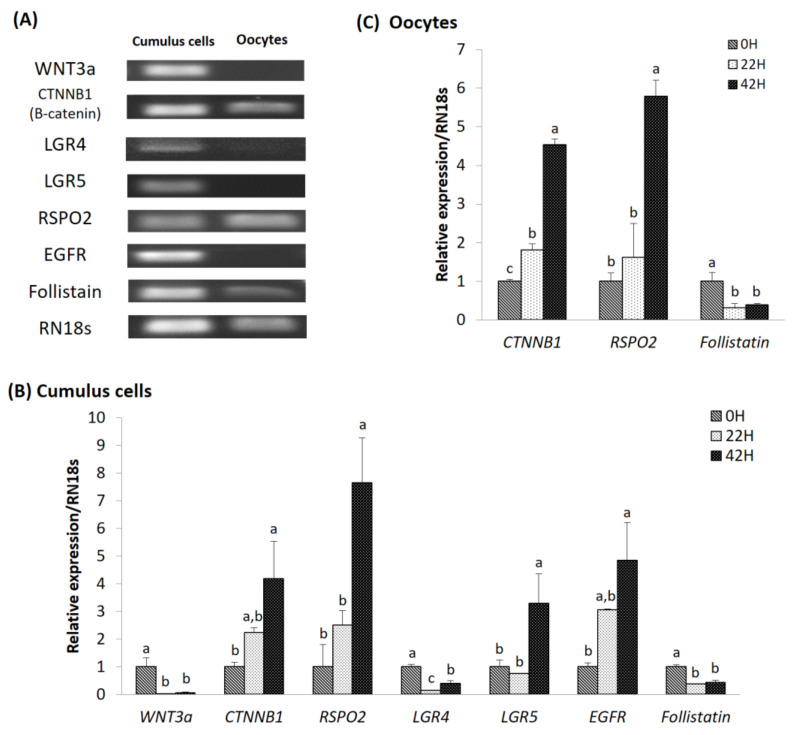
Identification and expression of RSPO2-associated genes under normal conditions in cumulus cells and oocytes. (**A**) Complementary DNA (cDNA) polymerase chain reaction analysis expression of *WNT3a*, *CTNNB1*, *RSPO2*, *LGR4*, *LGR5*, *EGFR*, and *Follistatin. RN18s* was used as a control. (**B**,**C**) Expression levels of genes relative to RSPO2 and WNT signaling were determined by Quantitative Polymerase Chain Reaction (qPCR). Mean ± standard error of the means (SEM) expression of *WNT3a*, *CTNNB1*, *RSPO2*, *LGR4*, *LGR5*, *EFGR*, and *Follistatin* mRNA in (**B**) cumulus cells and (**C**) oocytes. Each gene was isolated and statistically analyzed. Within each end point, bars with different letters (a, b and c) are significantly (*p* < 0.05) different. The experiment was replicated thrice.

We confirmed the expression of genes related to the RSPO2 and WNT signaling pathways over IVM time. In cumulus cells, the expression levels of *WNT3a*, *LGR4*, and *follistatin* were significantly lower in the 22 and 42 h groups than those in the 0 h group. Expression levels of *CTNNB1*, *LGR5*, *EGFR*, and *RSPO2* were significantly increased in the 42 h group compared to those in the 0 h group. In oocytes, expression levels of *follistatin* were significantly lower in the 22 and 42 h groups than in the 0 h group. Expression levels of *CTNNB1* and *RSPO2* were significantly increased in the 42 h group compared to those of the 0 h group. *WNT3a*, *EGFR*, *LGR4*, and *LGR5* expression were not detected (Figure 2).

### 3.3. Effect of RSPO2, WNT Inhibitor, or WNT Activator Treatment during IVM on Nuclear Maturation

Nuclear maturation experiments were performed with different concentrations of RSPO2 to determine the optimal concentration of treatment during IVM. The proportion of oocytes in the mature MII stage was increased in the groups treated with 100 ng/mL RSPO2 (86.5% ± 2.0%) compared with that of the control group (70.6% ± 4.1%) (Table 3); therefore, this concentration was used for subsequent experiments.

Comparison of the nuclear maturation rate under treatment with RSPO2 (100 ng/mL), WNT inhibitor (DKK1), or WNT activator (LiCl) during the IVM process showed that the MI ratio significantly increased and the MII ratio decreased in the LiCl-treated group (Table 4).

### 3.4. Effects of RSPO2, WNT Inhibitor, or WNT Activator Treatment on Gene Expression in Cumulus Cells and Oocytes during IVM

**Figure 3:**
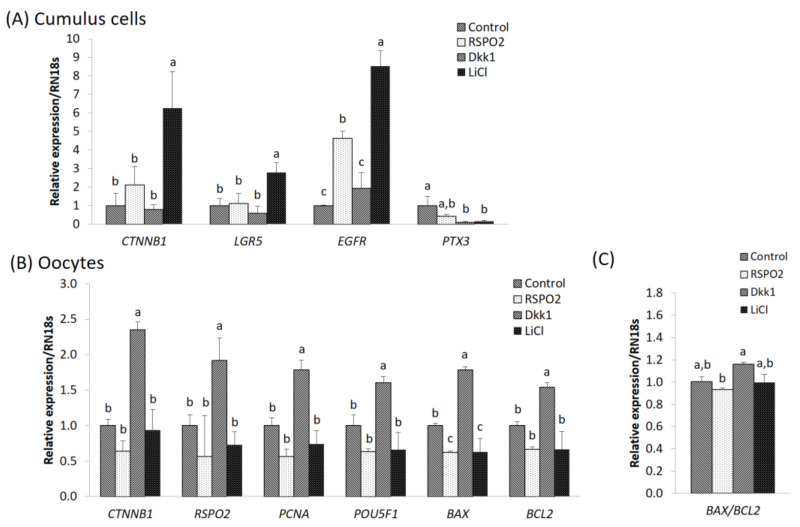
Expression levels of genes relative to RSPO2 and WNT signaling were determined by qPCR. Mean ± SEM expression of *CTNNB1*, *LGR5*, *PTX3*, *RSPO2*, *EGFR*, *PCNA*, *POU5F1*, *BAX,* and *BCL2* mRNA in (**A**) cumulus cells and (**B**,**C**) oocytes. Each gene was isolated and statistically analyzed. Within each end point, bars with different letters (a, b and c) are significantly (*p* < 0.05) different. The experiment was replicated thrice. SME: standard error of the means

In cumulus cells, mRNA expression levels of the RSPO2-WNT signaling pathway-related genes *CTNNB1*, *LGR5*, and *EGFR* were significantly increased in the LiCl treatment group compared to those of the control group. In addition, the expression level of *EGFR* was significantly increased in the RSPO2 and LiCl treatment groups compared to that of the control and DKK1 treatment groups. However, the RSPO2 treatment group showed significantly lower *EGFR* expression than that of the LiCl treatment group. In addition, the expression level of the cumulus cell expansion marker *PTX3* was significantly lower in the DKK1 and LiCl groups compared to that of the control group (Figure 3A).

In oocytes, expression levels of the RSPO2-WNT signaling pathway genes (*CTNNB1* and *RSPO2*) were significantly increased in the DKK1 treatment group compared to those of the control and LiCl treatment groups. Expression levels of cell proliferation (*PCNA*) or pluripotency marker (*POU5F1*) genes were significantly increased in the DKK1 treatment group compared to those of the control, RSPO2, and LiCl treatment groups. Expression of apoptosis-related genes (pro-apoptotic *BAX* or anti-apoptotic *BCL2*) were also significantly increased in the DKK1 treatment group compared to those of the control, RSPO2, and LiCl treatment groups (Figure 3B), and the *BAX*/*BCL2* ratio was significantly decreased in the RSPO2 treatment group than in the DKK1 treatment group (Figure 3).

### 3.5. Effect of RSPO2 on Embryo Development before Implantation

**Figure 4:**
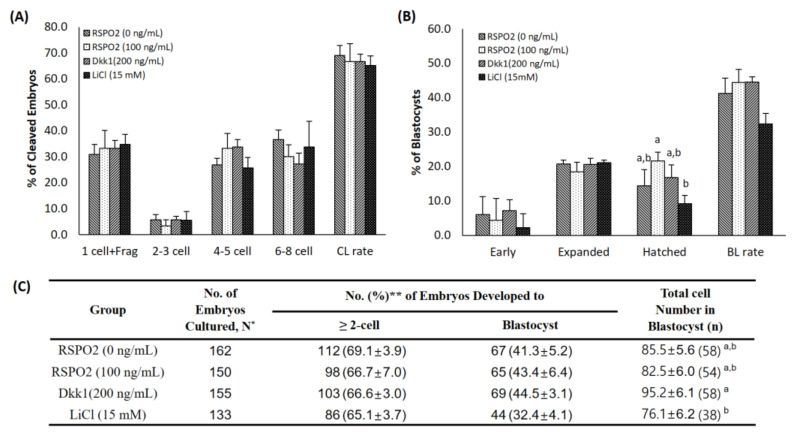
Effect of RSPO2, WNT inhibitor (Dkk1), or WNT activator (LiCl) treatment during IVM on embryonic development after in vitro fertilization (IVF) in terms of the (**A**) cleavage pattern and (**B**) blastocyst formation pattern of the IVF embryo. Within each end point, bars with different letters (a and b) are significantly (*p* < 0.05) different. CL, cleavage; BL, blastocyst. (**C**) Summary of embryonic development after IVF. The cleavage rate was measured on day 2, and the blastocyst formation rate was evaluated on day 7 of culture. * N: Four replications. RSPO2: R-Spondin2. ** Percentage of total embryos cultured.

The CL and BL formation rates of fertilized oocytes were evaluated 2 days and 7 days after fertilization, respectively. There were no significant differences between the control group and treatment groups (Figure 4). However, the number of hatched BLs significantly increased in the RSPO2 treatment group compared to that of the LiCl treatment group (Figure 4B). In addition, the total cell number in the LiCl treatment group was significantly lower than that in the DKK1 treatment group (Figure 4C).

### 3.6. Effect of RSPO2, WNT Inhibitor, or WNT Activator on mRNA Expression during Cumulus Cell Culture

**Figure 5:**
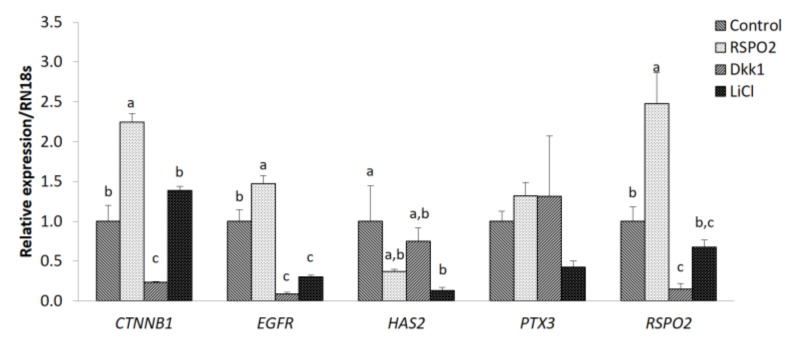
Expression levels of genes relative to cumulus cell expansion and RSPO2-WNT signaling were determined by qPCR. Mean ± SEM expression of *CTNNB1*, *EGFR*, *HAS2*, *PTX3*, and *RSPO2* mRNA in cumulus cells. Each gene was isolated and statistically analyzed. Within each end point, bars with different letters (a, b and c) are significantly (*p* < 0.05) different. The experiment was replicated thrice.

The effect of RSPO2 and WNT inhibitor or activator on primary cultured cumulus cells was analyzed to determine the level of mRNA expression of RSPO2-related genes in independent cumulus cells. The expression of *CTNNB1* was significantly increased in the RSPO2 treatment group compared to that of all other groups (control, DKK1, and LiCl treatment groups). However, the *CTNNB1* expression level was significantly decreased in the DKK1 treatment group compared to that of all other groups. The expression level of *EGFR* was significantly increased in the RSPO2 treatment group compared to that of all other groups. However, the *EGFR* level was significantly decreased in the DKK1 and LiCl treatment groups compared to those of the control and RSPO2 treatment groups. The expression level of *HAS2* was significantly decreased in the LiCl treatment group compared to that of the control group. The expression level of *RSPO2* was significantly increased in the RSPO2 treatment group compared to that of all other groups and was significantly decreased in the DKK1 treatment group compared to those of the control and RSPO2 treatment groups (Figure 5).

## 4. Discussion

The RSPO family of proteins has been reported to be associated with WNT signaling in tissues such as the intestinal stem cells and Paneth cells [43]. In addition, RSPO2 promotes proliferation and migration through the WNT/β-catenin pathway in human hepatocellular carcinoma [44] and positively regulates skeletal muscle formation [45]. In particular, RSPO2 has been shown to be involved in the maturation process of follicles via the WNT signaling pathway [6]. However, the role of RSPO2 and its interaction with WNT signaling in oocyte maturation has not been assessed beyond studies using rodents. In addition, RSPO2 has been reported to regulate WNT/β-catenin signaling through LGR4 and LGR5 [46]. In the present study, we first identified the expression and localization of RSPO2, CTNNB1, LGR4, and LGR5 in the porcine ovary and COCs. As a result, CTNNB1 was confirmed in ooplasm, and CTNNB1 and LGR4 were confirmed in granulosa cells. The expression of CTNNB1 (b-catenin) in oocytes is consistent with the results reported by Xie et al., 2008. [47]. In addition, the localization of RSPO2, LGR4 and LGR5 has been confirmed in the theca interna. These results imply that these factors play a major role in porcine follicular development.

We investigated the effect of RSPO2 on porcine cumulus cell and oocyte signaling during IVM. The results indicated that the major genes involved in RSPO2 and WNT signaling in porcine cumulus cells during IVM are *CTNNB1*, *LGR4*, *LGR5*, *EGFR,* and *RSPO2*. In a recent mouse study, it has been reported that oocyte-secreted RSPO2 promotes follicular development by activating WNT/CTNNB1 signaling in granulosa cells [3]. Our results indicate that RSPO2 activates CTNNB1 signaling through LGR4 or LGR5 in porcine. Consistent with our findings that *LGR4* and *follistatin* mRNA expression levels were both reduced during IVM, a previous study showed that RSPO2-LGR4 signaling in skeletal myogenesis modulated the *follistatin* gene through the WNT/β-catenin pathway [48]. Another study showed that LGR4 deficiency reduced the strength of the WNT-EGFR-ERK signal, which negatively affected granulosa-lutein cell differentiation and corpus luteum maturation [36]. By contrast, the EGFR-ERK signal is essential for oocyte maturation, ovulation, and luteinization, and *EGFR* expression significantly increased in our study during IVM. This suggests interactions among several factors present in the IVM medium. There is evidence that the expression of *EGFR* in cumulus cells is essential for EGFR signaling from mural granulosa cells. For example, EGFR ligands, AREG, EREG, BTC, and EGF, stimulate the maturation of oocytes in the COC during the IVM process but do not stimulate denuded oocytes that remove the surrounding cumulus cells [49,50]. These results suggest that EGFR plays a major role in cumulus cells.

Treatment with the WNT activator LiCl showed that the MI ratio was high and the MII rate was low. Thereafter, IVF was performed by using mature oocytes, showing no significant difference between CL and BL rates, although the number of hatched BLs significantly increased in the RSPO2 treatment group compared to that of the LiCl treatment group, and the total cell number in the LiCl treatment group was significantly lower than that in the DKK1 (WNT inhibitor) treatment group. Previous studies have reported that the WNT/β-catenin pathway has no beneficial effect on oocyte growth, and that the pathway was not activated during embryonic development before implantation; the other study showed that treatment with DKK1 during the IVM of porcine oocytes increased the MII rate and the number of BLs [27,51]. Although no increase in the number of BLs was observed in the DKK1-treated group, the total cell number in BLs significantly increased compared with the LiCl-treated group. This shows that the quality of BLs was improved with the inhibition of WNT signaling.

Next, matured cumulus cells or oocytes were assessed at the mRNA level. In cumulus cells, genes related to the RSPO2-WNT signaling pathway (*CTNNB1*, *LGR5*, *EGFR*) were significantly increased in the LiCl treatment group compared to those of all other treatment groups, and the level of the cumulus cell expansion marker *PTX3* was significantly decreased in the DKK1 and LiCl treatment groups compared to that of the control group. Previous studies have shown that RSPO2 is derived from oocytes and promotes follicular growth [6]. In addition, RSPO2 promoted WNT signaling through LGR4 and LGR5 in HEK293 cells [46]. Moreover, RSPO2 was shown to inhibit the growth of colorectal tumors through LGR5-dependent WNT signaling [52]. However, in the present study, the expression level of *EGFR* was significantly increased in the RSPO2 and LiCl treatment groups compared with that of the control and DKK1 treatment groups (Figure 3A). This suggests that RSPO2 increases the expression of *EGFR* in a WNT pathway-independent manner. Similarly, a previous study found increased CTNNB1 levels under treatment with RSPO2 and DKK1 compared to those of the WNT3a treatment group [4].

In oocytes, the expression levels of *CTNNB1*, *RSPO2*, *PCNA*, *POU5F1*, *BAX*, and *BCL2* were significantly increased in the DKK1 treatment group compared to those of all other groups. Although CTNNB1 may improve the activities of FSH and LH in the antral follicles, excessive activation of CTNNB1 has been reported to have a negative effect on ovulation and luteinization by LH [7]. In addition, the *BAX/BCL2* ratio was higher in the DKK1 treatment group, and apoptosis was relatively more frequent compared with the RSPO2 treatment group, whereas RSPO2 treatment increased the expression level of *EGFR* in the cumulus cells, suggesting that treatment with RSPO2 reduced apoptosis in oocytes.

The above experiments could not exclude the potential role of other factors in controlling *EGFR* expression. In mouse studies, for example, oocyte-derived paracrine factors, especially GDF9 and BMP15, promote *EGFR* expression in cumulus cells prior to LH surge. Through this, the cumulus cells are programmed to receive LH-induced EGF-like peptide signals from granulosa cells [53]. Therefore, excluding the role of other factors in the IVM process, primary cultured cumulus cells were used to focus only on the interaction between RSPO2 and cumulus cells. After 42 h of culture, expression levels of *CTNNB1*, *EGFR*, and *RSPO2* were significantly increased after RSPO2 treatment compared to those of the other groups, which is consistent with the increase in *CTNNB1* levels upon RSPO2 treatment in HEK293 cells [4]. However, these results are different from qPCR data of the cumulus cells obtained after IVM. This is thought to be the effect of EGF, which is essential for oocyte maturation, and other factors derived from oocytes. Therefore, the results obtained by culturing only the cumulus cells other than these factors might be more reliable. Previous mouse studies have also shown that oocyte-derived RSPO2 promotes follicular development [6]. In addition, CTNNB1 promotes follicular development before ovulation but inhibits LH-mediated ovulation and lutein formation [7]. Activation of the EGFR signal in mature follicle granulosa cells is essential for oocyte maturation, ovulation, and luteinization [54]. Therefore, RSPO2 appears to be one of the key factors controlling the expression of *CTNNB1* and *EGFR* in the IVM process, which in turn regulates oocyte maturation, ovulation, and luteinization.

## 5. Conclusions

This study is the first to confirm the localization of RSPO2, CTNNB1, LGR4, and LGR5 in porcine ovary and to confirm the effect of RSPO2 in the porcine IVM process. Similar to recent mouse studies reporting that RSPO2 promotes follicular development by activating WNT/CTNNB1 signaling in granulation cells, our results suggest that these factors play an important role in porcine follicle development. In addition, RSPO2 is expected to contribute to the maturation of oocytes by modulating WNT/CTNNB1 signaling during the maturation of oocytes. Taken together, these results suggest that the RSPO2 and WNT/CTNNB1 signaling pathways are required for porcine follicle development and are involved in the EGFR-ERK signaling pathway.

## Figures and Tables

**Table 1:** Antibodies used for immunofluorescence staining.

Name	Host	Dilution	Cat #
Anti-RSPO2	Mouse	1:200	MABS1709
Anti-CTNNB1	Rabbit	1:200	PA5-19469
Anti-LGR4	Rabbit	1:200	ab137480
Anti-LGR5	Mouse	1:200	ab273092
Alexa Fluor 594	Anti-Mouse	1:400	A11032
Alexa Fluor 594	Anti- Rabbit	1:400	A21207

**Table 2:** Primer sequences for gene expression analysis.

mRNA	Primer Sequences	Product Size (bp)	GenBank Accession Number
*RN18s*	F: 5′-CGCGGTTCTATTTTGTTGGT-3′ R: 5′-AGTCGGCATCGTTTATGGTC-3′	219	AY265350
*LGR4*	F: 5′-GTGGGAGGGATTTATTTACAG-3′ R: 5′-TGAATGCAGTGAAAGTACTCAG-3′	188	XM_013994475
*LGR5*	F: 5′-CAAGATCCAAACACACAAGC-3′ R: 5′-TAGAGACATGGGACAAATGC-3′	199	KP717080
*RSPO2*	F: 5′-GCTTTGAGGAATGTCCAGAT-3′ R: 5′-TGGTTGGACATGGTATCGTA-3′	198	NM_001293141
*Follistatin*	F: 5′-TCCTGTGAAGACATCCAGTG-3′ F: 5′-CTTTACTTCCAGCAGCACAC-3′	204	NM_001003662
*Wnt3a*	F: 5′-CAGCCAGACTTCTCCTCACT-3′ F: 5′-TGGTGGATATAGCAGCATCA-3′	220	XM_003123621
*B-catenin* (CTNNB1)	F: 5′-CAATGGCTTGGAATGAGACT-3′ F: 5′-CAGCCCATCAACTGGATAGT-3′	200	NM_214367
*EGFR*	F: 5′-ATCGGTTTAGGCTACTCACG-3′ R: 5′-GCACAAGGCTGTCCTTATTT-3′	193	NM_214007
*Has2*	F: 5′-TTACAATCCTCCTGGGTGGT-3′ R: 5′-TCAAGCACCATGTCGTACTG-3′	199	NM_214053
*PTX3*	F: 5′-AGACTTTATGCCATGGTGCT-3′ R: 5′-TGACAGTGAGCAATGAACAA-3′	195	NM_001244783
*PCNA*	F: 5′-CCTGTGCAAAAGATGGAGTG-3′ R: 5′-GGAGAGAGTGGAGTGGCTTT-3′	187	XM_003359883
*POU5F1*	F: 5′-GCGGACAAGTATCGAGAACC-3′ R: 5′-CCTCAAAATCCTCTCGTTGC-3′	200	NM_001113060
*BAX*	F: 5′-TGCCTCAGGATGCATCTACC-3′ R: 5′-AAGTAGAAAAGCGCGACCAC-3′	199	XM_003127290
*BCL2*	F: 5′-AATGACCACCTAGAGCCTTG-3′ R: 5′-GGTCATTTCCGACTGAAGAG-3′	182	NM_214285

F: Forward, R: Reverse.

**Table 3:** Effect of R-spondin2 (RSPO2) treatment during in vitro maturation (IVM) on nuclear maturation.

Group	Oocytes Cultured for Maturation, N *	Number of Oocytes at the Stage of							
		Germinal Vesicle (%)		Metaphase I (%)		Anaphase and Telophase I (%)		Metaphase II (%)	
RSPO2 (0 ng/mL)	211	29	(13.7 ± 2.5)	16	(7.63 ± 3.0)	17	(8.1 ± 1.0) ^a,b^	149	(70.6 ± 4.1) ^a^
RSPO2 (0.5 ng/mL)	203	20	(9.9 ± 1.7)	16	(7.9 ± 2.9)	17	(8.4 ± 0.9) ^a^	150	(73.9 ± 5.0) ^a^
RSPO2 (5 ng/mL)	204	21	(10.3 ± 1.0)	8	(3.9 ± 2.5)	13	(6.4 ± 1.6) ^a,b^	162	(79.4 ± 1.3) ^a,b^
RSPO2 (10 ng/mL)	199	21	(10.6 ± 3.3)	11	(5.5 ± 2.8)	9	(4.5 ± 0.5) ^b^	158	(79.4 ± 5.8) ^a,b^
RSPO2 (100 ng/mL)	207	14	(6.8 ± 1.3)	2	(1.0 ± 0.5)	12	(5.8 ± 1.3) ^a,b^	179	(86.5 ± 2.0) ^b^

^a,b^ Values with different superscript letters within a column differ significantly (*p* < 0.05). * Replicated four times.

**Table 4:** Effect of R-spondin2 (RSPO2), WNT inhibitor, or activator treatment during IVM on nuclear maturation.

Group	Oocytes Cultured for Maturation, N *	Number of Oocytes at the Stage of							
		Germinal Vesicle (%)		Metaphase I (%)		Anaphase and Telophase I (%)		Metaphase II (%)	
RSPO2 (0 ng/mL)	183	7	(3.8 ± 1.4)	8	(4.4 ± 1.0) ^b,c^	5	(2.7±1.1) ^a^	163	(89.1±1.3) ^b^
RSPO2 (100 ng/mL)	179	5	(2.8 ± 0.5)	1	(0.6 ± 0.6) ^a^	5	(2.8±1.1) ^a^	168	(93.9±1.2) ^c^
Dkk1 (200 ng/mL)	182	7	(3.8 ± 1.0)	3	(1.6 ± 0.6) ^a,b^	10	(5.5±0.6) ^a,b^	162	(89.0±1.8) ^b^
LiCl (15 mM)	185	9	(4.9 ± 1.3)	13	(7.0 ± 1.6) ^c^	11	(5.9±0.7) ^b^	152	(82.2±0.8) ^a^

^a,b,c^ Values with different superscript letters within a column differ significantly (*p* < 0.05). * Replicated four times.

## Data Availability

The data presented in this study are available on request from the corresponding author.
